# Physiological and transcriptome analyses of *Opisthopappus taihangensis* in response to drought stress

**DOI:** 10.1186/s13578-019-0318-7

**Published:** 2019-07-04

**Authors:** Huihui Gu, Yan Yang, Minghui Xing, Caipeng Yue, Fang Wei, Yanjie Zhang, Wenen Zhao, Jinyong Huang

**Affiliations:** 1 School of Chemical Engineering and Energy, Zhengzhou University, Zhengzhou, Henan 450001 People’s Republic of China; 2 School of Life Sciences, Zhengzhou University, Zhengzhou, Henan 450001 People’s Republic of China; 3 School of Agricultural Sciences, Zhengzhou University, Zhengzhou, Henan 450001 People’s Republic of China

**Keywords:** Drought stress, Transcriptome, Physiological analyses, *Opisthopappus taihangensis*, RNA-Seq

## Abstract

**Background:**

Water scarcity is considered to be a severe environmental constraint to plant survival and productivity. Studies on drought-tolerant plants would definitely promote a better understanding of the regulatory mechanism lying behind the adaptive response of plants to drought. *Opisthopappus taihangensis* (ling) shih is a typical drought-tolerant perennial plant species endemically distributed across the Taihang Mountains in China, but the underlying mechanism for drought tolerance of this particular species remains elusive.

**Results:**

To mimic natural drought stress, *O. taihangensis* plants were treated with two different concentrations (25% and 5%) of polyethylene glycol (PEG6000), which represent the H group (high salinity) and the L group (low salinity), respectively. The physiological characteristics of these two groups of plants, including relative water content maintenance (RWC), proline content and chlorophyll content were assessed and compared with plants in the control group (CK), which had normal irrigation. There was not a significant difference in RWC when comparing plants in the L group with the control group. Proline was accumulated to a higher level, and chlorophyll content was decreased slightly in plants under low drought stress. In plants from the H group, a lower RWC was observed. Proline was accumulated to an even higher level when compared with plants from the L group, and chlorophyll content was further reduced in plants under high drought stress. Transcriptomic analysis was carried out to look for genes that are differentially expressed (DEGs) in *O. taihangensis* plants coping adaptively with the two levels of drought stress. A total of 23,056 genes are differentially expressed between CK and L, among which 12,180 genes are up-regulated and 10,876 genes are down-regulated. Between H and L, 6182 genes are up-regulated and 1850 genes are down-regulated, which gives a total of 8032 genes. The highest number of genes, that are differentially expressed, was obtained when a comparison was made between CK and H. A total of 43,074 genes were found to be differentially expressed with 26,977 genes up-regulated and 16,097 genes down-regulated. Further analysis of these genes suggests that many of the up-regulated genes are enriched in pathways involved in amino acid metabolism. Besides, 39 transcription factors (TFs) were found to be continuously up-regulated with the increase of drought stress level.

**Conclusion:**

Taken together, the results indicate that *O. taihangensis* plants are able to live adaptively under drought stress by responding physiologically and regulating the expression of a substantial number of drought-responsive genes and TFs to avoid adverse effects.

**Electronic supplementary material:**

The online version of this article (10.1186/s13578-019-0318-7) contains supplementary material, which is available to authorized users.

## Background

Plants in the field are facing a lot of environmental challenges, and drought is a major abiotic stress, which can have negative effects on global food security and agricultural production [1]. Water scarcity is an inevitable result of climate change, especially global warming. However, the demand for water for non-agricultural use is increasing drastically every year, and this has posed a serious challenge to agricultural production worldwide. It is therefore important for molecular breeders to understand the resistance mechanism of plants to drought and to be able to utilize this knowledge to breed for high drought tolerance and recoverability [2]. Plants are able to sense environmental changes, to generate and transduce perceptual signals, and to modify their physiology by regulating the expression of various regulatory and functional genes in an integrated and sophisticated manner [3, 4]. In response to drought, plants can alter the expression of various genes. Based on their putative functions, these genes can be sub-divided into two major groups [5]. Genes that function in transcriptional regulation and signaling cascades, including transcription factors (TFs), phytohormones, phosphatases and protein kinases, constitute the first group. The second group consists of genes that protect plant cells against stresses, such as heat shock proteins (HSPs), dehydrins, senescence-related genes, membrane protectants, osmoprotectants, transporters, antioxidants and so on. During drought, it is very easy for plants to suffer from dehydration of its cells. One of the major protective adaptations that plants exhibit under dehydration is to promote the production of plant hormones, including auxin, ABA (abscisic acid), JA (jasmonic acid), GA (gibberellic acid), ET (ethylene) and BR (brassinosteroid), and these plant hormones also contribute to stress tolerance in plants [6].

Previous studies have shown that the expression of a significant number of transcription factors (TFs) can be induced by drought, and that those TFs are involved in plant protective adaptations and drought resistance. Members of some TF families, including bHLH and MYB [7], WRKY [8], AP2-EREBP [9] and NAC [10], have been shown to be involved in the stress-induced signaling cascade. Recently, a substantial number of stress-inducible genes have been identified using microarray analysis in different plant species, such as *Arabidopsis*, barley, rice and grape [11–14].

RNA-Seq is a recently developed tool, which can be used for transcriptome profiling. Compared with the conventional method, this method has many advantages: it can still work when there is no genomic sequence data available; it is high-throughput, and it has relatively low background noise [15]. Transcriptome analysis has been widely used to determine expression profiles and gene structures in many plant species under stress [16, 17]. Transcriptome data have also been used to identify genes, which regulate the complex interaction and metabolic processes of plants under drought stress [18, 19]. The use of this technology allows researchers to identify candidate genes, which are responsible for plant adaptive response to drought, and to breed plants for high drought resistance and recoverability. To the best of our knowledge, the drought response of *Opisthopappus taihangensis* has not been studied in detail so far. In this study, RNA-Seq was used to analyze differential gene expression in leaves of *O. taihangensis* under drought stress, and the reliability of the comparative transcriptome data was further validated with qRT-PCR.

*Opisthopappus taihangensis* (Ling) Shih, which belongs to the family Asteraceae, is an endangered plant species, and it is endemic to the Taihang Mountains (China) across Henan, Hebei and Shanxi provinces. *Opisthopappus taihangensis* plants are found on the cracks of the steep cliffs or on the slopes at an elevation of about 1000 m. *Opisthopappus taihangensis* have been severely picked by people for its medicinal and ornamental values. Its distribution range is also decreasing due to changes in their habitat. As a result, *O*. *taihangensis* has been listed among the Class II State-Protected Endangered Plant Species [20, 21]. In response to some of the challenges imposed by its living environment, *O. taihangensis* has evolved some adaptive traits, such as drought tolerance and cold temperature endurance. The purpose of this study is to identify drought-responsive genes, and to deeply clarify the signaling, regulatory and metabolic mechanisms that operate under drought stress.

## Materials and methods

### Plant material and drought treatments

*Opisthopappus taihangensis* used in this study were grown in temperature-controlled incubators with a 16-h light (25 °C)/8-h dark (20 °C) photoperiod, and a humidity of 60%. Water-deficit stress was performed with polyethylene glycol (PEG 6000): 30-day-old plants were transplanted to 0% (control group, CK), 5% (low salinity, L) and 25% (high salinity, H) PEG 6000. For each treatment, there were three biological replications. Fresh leaves were collected 2 days after the stress treatments, flash frozen and kept in liquid nitrogen until use.

### Measurement of chlorophyll content

To determine the chlorophyll content in collected samples, harvested leaves were freeze-dried for 48 h, and were ground to a fine powder with liquid nitrogen. The lyophilized samples (50 mg) were transferred to a 10 mL of the extraction mixture (50% ethanol and 50% acetone) and mixed for 10 min, followed by sonication for 30 min, and the mixtures were then placed at 4 °C for 12 h. The samples were centrifuged with a cooled centrifuge (4 °C) at 5000 rpm for 10 min, and the supernatants were collected and used for spectrophotometry analysis (Shimadzu, Japan) at two wavelengths: 645 nm and 663 nm. The total chlorophyll content in each sample was represented as the mean of three biological replicates. The chlorophyll content was calculated using the following formula: chlorophyll content (mg/g) = 8.04 * A_645_ + 20.29 * A_663_ [22].

### Determination of relative water content (RWC)

To measure RWC in each sample, the fresh weight (FW) of detached leaves were measured immediately. Leaves were rehydrated in water for 24 h until fully turgid to determine the turgid weight (TW). Subsequently, samples were dried in an oven at 65 °C for 48 h, and the dry weight (DW) was measured and recorded. RWC was calculated using the following formula: RWC (%) = (FW − DW)/(TW − DW) × 100. Three biological replicates were performed to ensure the accuracy of the test.

### Analysis of proline content

Proline content was measured following the protocol described by Bates LS [23]. The absorbance of samples was measured at 520 nm, and the concentration of proline in each sample was calculated using the following formula with l-proline as a standard: proline (μg/g) = (C * V)/W, where C is the proline concentration in a given sample, V is the total volume of the sample, and W is the dry weight of the sample. Three biological replicates were performed for all drought treatments.

### RNA extraction and RNA sequencing

Total RNA was extracted from leaves using the Plant RNA Kit (OMEGA, USA) according to the manufacturers’ instructions. The quantity and quality of total RNA were assessed using the NanoPhotometer^®^ spectrophotometer (IMPLEN, CA, USA). RNA samples were isolated from the three control groups (CK1, CK2, CK3), low stress (L1, L2, L3) and high stress (H1, H2, H3) for the construction of RNA-Seq libraries, which were analyzed by Novogene (Beijing, China) on an Illumina HiSeq 2500 platform. The raw data were transformed into sequencing information by base calling and stored as FastQ format files. The clean and filtered reads were aligned to the references genome of *Arabidopsis thaliana* using TopHat2.

### Expression validation by qRT-PCR

The RNA samples of drought-stressed leaf were used for qRT-PCR analysis following the method described in previous studies [24]. Gene-specific qRT-PCR primers were designed using the Primer 5 software and listed in Additional file 1: Table S1. The qRT-PCR analysis was performed using the LightCycler Multiplex DNA Master Kit (Roche, Switzerland) with a 10-μL reaction mixture (5 μL of 5× reaction mix, 3 μL of distilled water, 1 μL of cDNA and 0.5 μL of 10 mM each primer) and performed on LightCycler 480 Real-Time PCR System (Roche, Switzerland) using the following program: 95 °C for 2 min, 45 cycles of 95 °C for 10 s, 56 °C for 15 s, and 72 °C for 10 s. Three biological replicates were performed for each of the selected genes, and the expression levels of all relative gene were calculated using the 2^−△△CT^.

### Differential expression analysis

Differential expression analysis was performed using the DESeq R package (1.10.1). The resulting P values were adjusted using the Benjamini and Hochberg’s approach to control the false discovery rate (FDR). Genes with an adjusted P-value < 0.05 picked up by DESeq were assigned as differentially expressed. *P* value was adjusted using q value [25]. *q* value < 0.005 and |log2(fold change)| > 1 was set as the threshold for significantly differential expression.

### Gene functional annotation

To predict the possible functions of the differentially expressed genes (DEGs) and the potential biological pathways in which they are involved, DEGs were annotated using the following databases: Nr (NCBI non-redundant protein sequences); Nt (NCBI non-redundant nucleotide sequences); Pfam (Protein family); KOG/COG (Clusters of Orthologous Groups of proteins); Swiss-Prot (A manually annotated and reviewed protein sequence database); KO (KEGG Ortholog database) and GO (Gene Ontology). GO enrichment analysis of the DEGs was performed with the GOseq R packages based on Wallenius non-central hypergeometric distribution.

### Statistical analysis

To ensure the accuracy of the experiment, three biological replications were applied to all tests. Statistical analyses were performed using the one-way analysis of variance (ANOVA) followed by Duncan’s tests with SPSS version 21.0 (SPSS, Chicago, IL, USA). A significance level of P < 0.05 or P < 0.01 was applied.

## Results

### Analysis of physiological changes of *Opisthopappus taihangensis* under drought stress

**Fig. 1:**
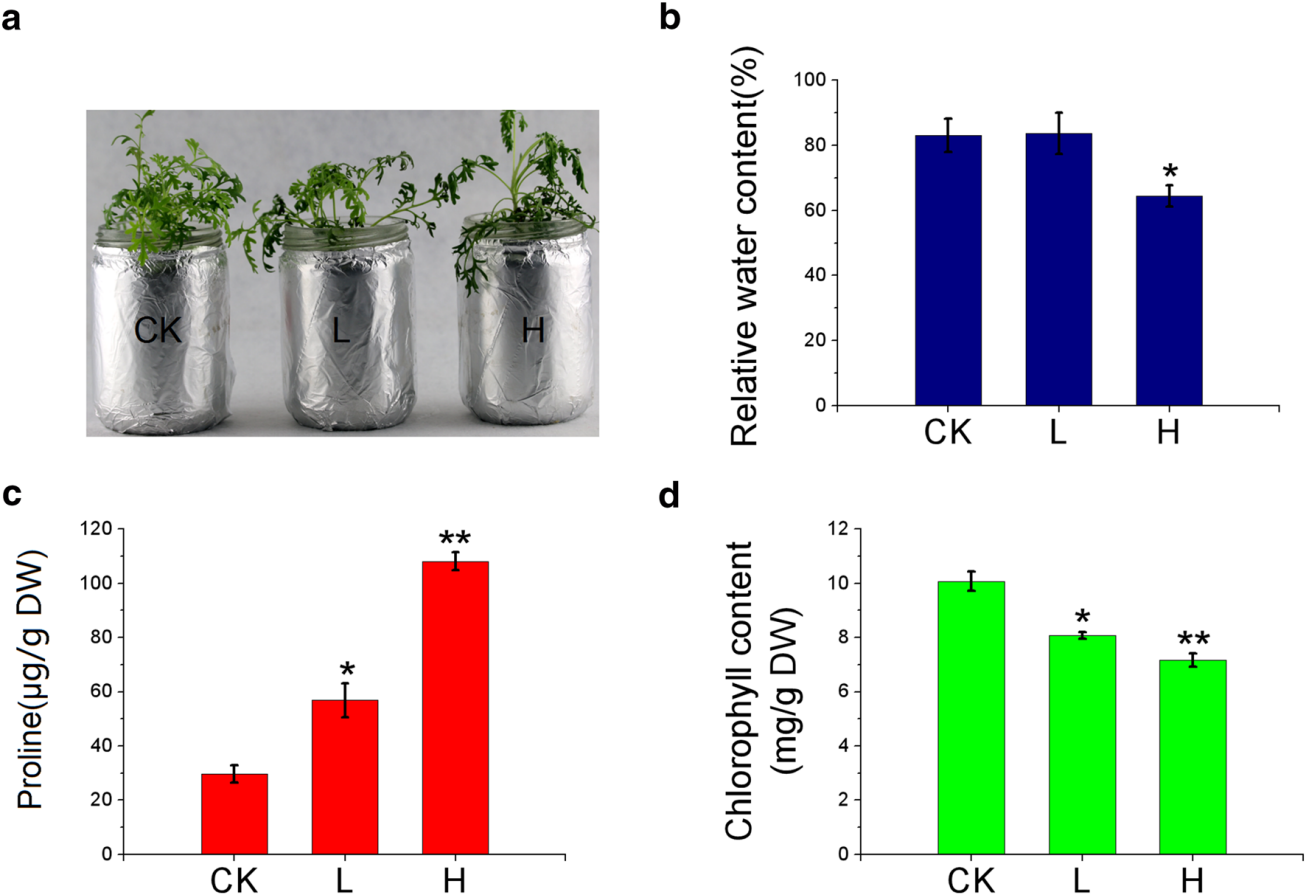
Analyses of physiological indexes (**a** morphology, **b** RWC, **c** proline content and **d** chlorophyll content) in *Opisthopappus taihangensis* under different drought-stress conditions. Error bars represent the standard error of the mean (n = 3). Statistical significance of the differences between different drought treatments was determined with one-way ANOVA. *P < 0.05 and **P < 0.01. The results were analyzed in triplicates

To investigate the physiological response of *O. taihangensis* under drought stress, plants were treated with two different PEG concentrations. The results indicate that high drought stress could cause a morphological change to plants: plants were severely withered under high-stress condition (Fig. 1a). In contrast, no significant difference was observed between the control group and the low-stress group in terms of the level of witheredness. This suggests that controlled growth was an adaptive response in plants to cope with drought stress. Consistent with the phenotype, the RWC of leaves in *O. taihangensis* under low drought stress was similar to that of the control group. However, RWC dropped significantly by 16.7% in plants under the high drought stress (Fig. 1b). It is therefore supported that *O. taihangensis* is able to maintain a sufficient amount of water under mild drought stress to allow them to cope with water scarcity.

Furthermore, with the increase of PEG concentration, the contents of proline in drought stress treatment groups were increased gradually compared to that of the control groups (Fig. 1c). The result indicated that *O*.* taihangensis* could improve plant drought tolerance by accumulating proline. In addition, the chlorophyll contents of the plants from the two stress groups were decreased slightly compared to that of the control group (Fig. 1d), which suggests that *O*.* taihangensis* is still able to carry out photosynthesis even under drought stress.

### Characterization of RNA-Seq

To study the drought resistance mechanism in *O. taihangensis* and to identify candidate genes involved in drought stress, deep RNA sequencing of the *O. taihangensis* leaves subjected to different degrees of drought stress was carried out using the Illumina sequencing platform. The data of *O. taihangensis* has been uploaded to the NCBI SRA database with an Accession Number of PRJNA526138. Approximately, 71.50 GB of clean reads data were obtained with a Q30 ≥ 91.76 and GC content between 42.35 and 43.14%, and the data was considered to be highly reliable for further study (Table 1).

**Table 1:** The statistical summary of the de novo assemblies for CK1, CK2, CK3, L1, L2, L3, H1, H2 and H3

Sample	Raw reads	Clean reads	Clean bases	Error (%)	Q20 (%)	Q30 (%)	GC content (%)	Total mapped
CK1	49,312,122	45,107,380	6.77G	0.03	97.78	93.77	42.54	36,887,606 (81.78%)
CK2	48,293,982	44,263,796	6.64G	0.03	97.81	93.88	42.42	36,269,274 (81.94%)
CK3	48,332,046	44,127,512	6.62G	0.03	97.45	93.07	42.35	35,929,572 (81.42%)
L1	62,783,092	61,707,438	9.26G	0.03	96.89	91.76	43.01	49,664,802 (80.48%)
L2	66,647,822	65,857,376	9.88G	0.03	97.07	92.11	42.80	53,191,524 (80.77%)
L3	61,247,186	60,318,434	9.05G	0.03	97.11	92.19	42.76	48,581,740 (80.54%)
H1	49,581,626	48,545,588	7.28G	0.03	97.34	92.74	43.14	39,548,036 (81.47%)
H2	51,319,342	50,154,068	7.52G	0.03	97.34	92.75	43.10	40,928,638 (81.61%)
H3	57,660,540	56,558,028	8.48G	0.03	97.17	92.38	43.13	45,881,730 (81.12%)

**Fig. 2:**
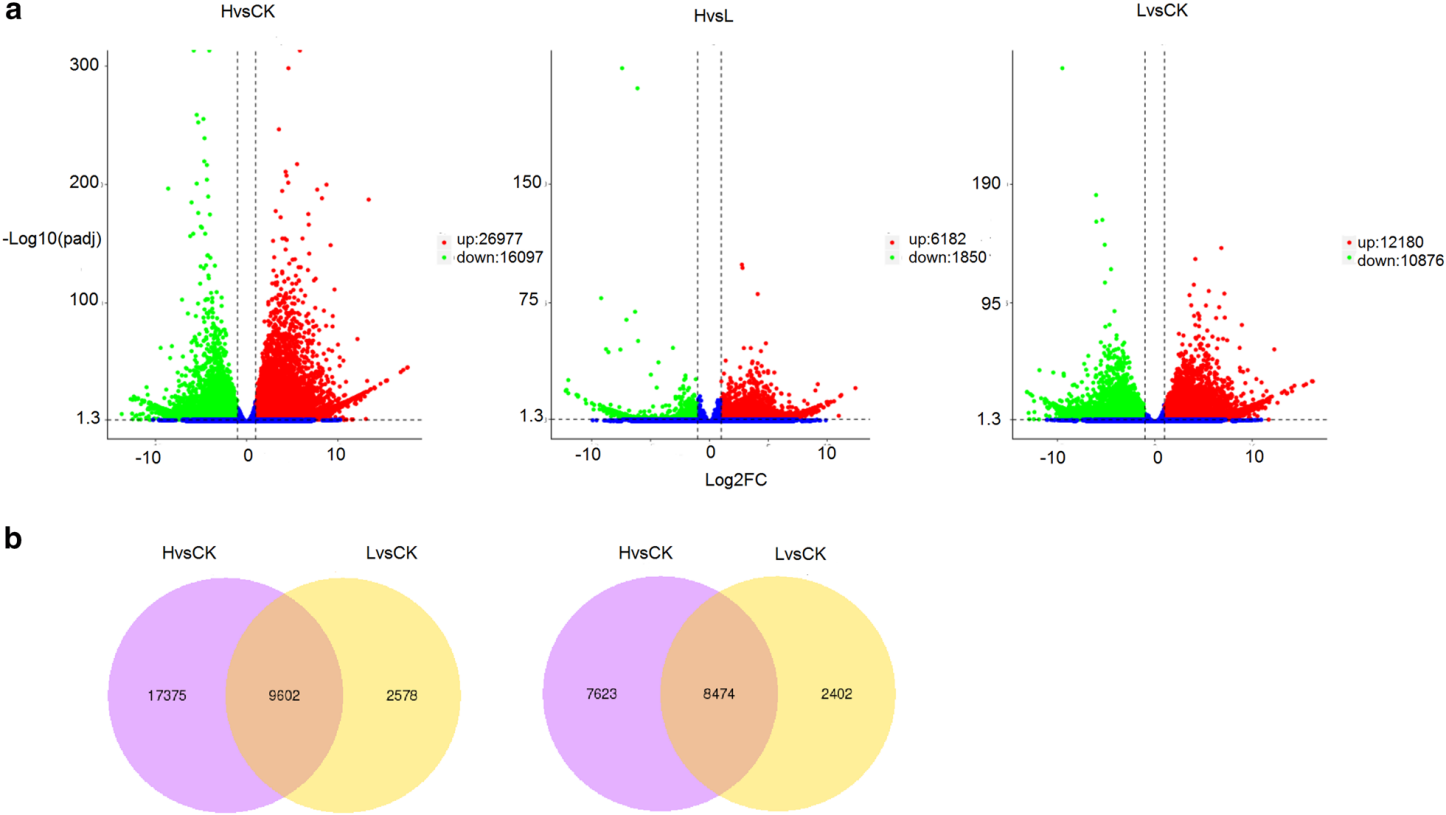
Verification and analysis of differentially expressed genes in response to drought treatments. **a** Volcanic plots of differentially expressed genes in H vs CK, H vs L and Lv CK. Blue dots represent genes that showed no response to drought stress. Up-regulated and down-regulated genes are represented as red and blue dots, respectively. **b** The number of common and specific differentially expressed genes among the three groups in the Venn diagram

The DEGs were assigned when the following criteria were satisfied: the log2 Fold Change > 1, and false discovery rate (FDR) < 0.05. Volcano plots show that 26,977 genes are up-regulated, and that 16,097 genes are down-regulated between H and CK. Between H and L, 6182 genes are up-regulated and 1850 genes are down-regulated (Fig. 2a). In addition, 12,180 genes were found to be up-regulated, and 10,876 genes were down-regulated between L and CK. The Venn diagram visually shows the number of common and specific DEGs among the three groups. Notably, 9602 and 8474 genes were found to be up-regulated and down-regulated, respectively, in both stress group compared to the control group (Fig. 2b), and these common DEGs probably play important roles in drought tolerance in *O. taihangensis*.

**Fig. 3:**
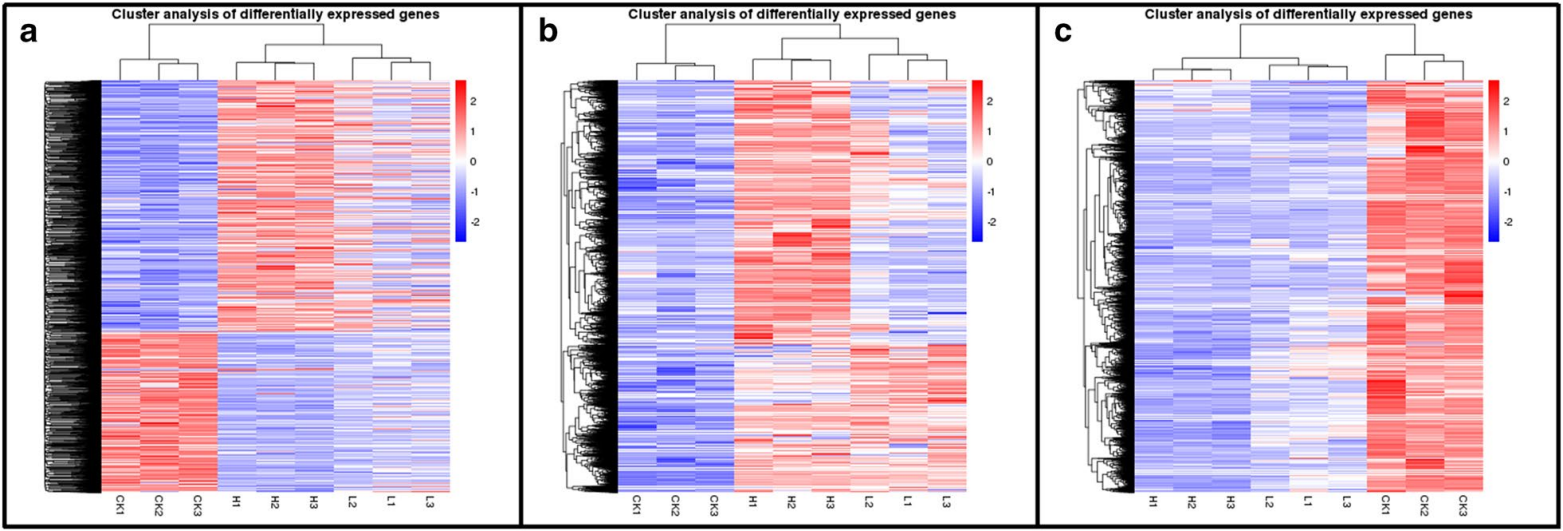
Hierarchical clustering of the DEGs (**a**) in response to drought. log10FPKM values are represented as colors ranging from blue (− 2: low expression) to red (2: highest expression). Each column represents a library, each row represents a DEG. The color bar on the left indicates the range of the highest log10FPKM value within nine libraries for each gene. **b** Clustering analysis of the all the up-regulated DEGs in the libraries of H, L and CK. **c** Clustering analysis of all the down-regulated DEGs in the libraries of H, L and CK

The expression patterns of all DEGs in three drought treatments were further analyzed, and the hierarchical clustering of the DEGs in H, L and CK were shown in Fig. 3. In comparison with CK, a large number of genes were differentially expressed in the L group. These genes were more significantly differentially expressed in the H group when compared with the CK group (Fig. 3a). The cluster analysis of all the up-regulated (Fig. 3b) and down-regulated DEGs (Fig. 3c) showed similar results. The higher the stress level is, the more significantly differentially expressed the stress-resistance genes are. These results suggest that plants can cope with drought by increasing or decreasing the expression of massive drought-responsive genes to different levels in response to different stress levels to maintain plants growth, development and survival.

### Functional classification of drought-responsive genes by GO analysis

**Fig. 4:**
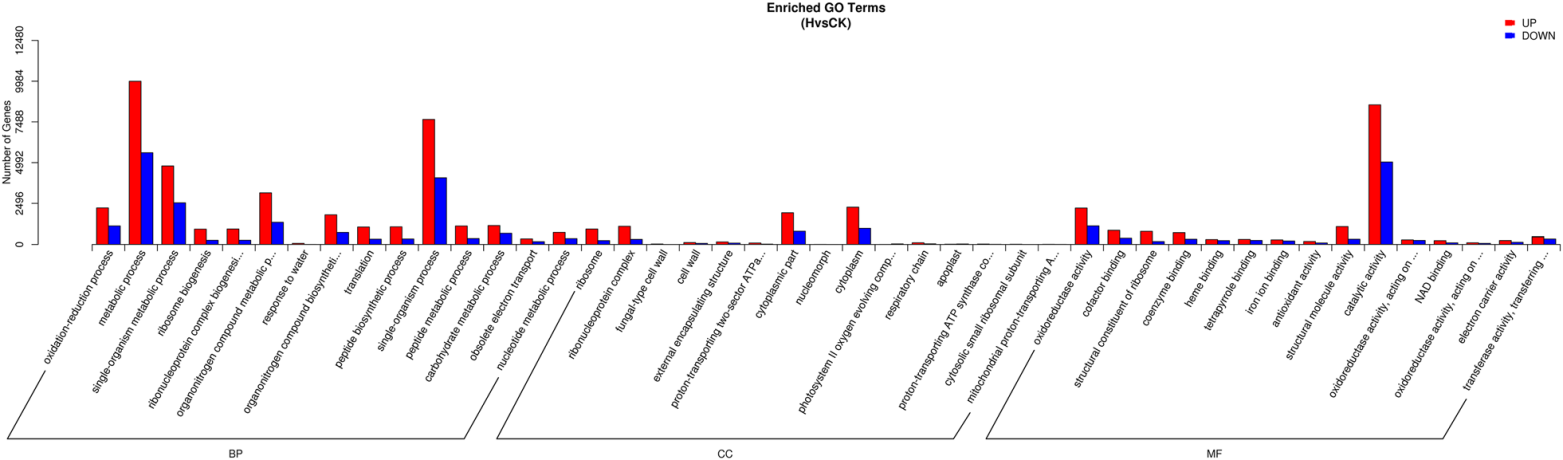
GO classifications of DEGs between the H and the CK groups. The Y-axis represents the number of genes in a category. The number of up-regulated genes is represented by the size of the red bars whereas the number of down-regulated genes is represented by the size of the blue bars. *BP* biological process, *CC* cellular components, *MF* molecular function

Because the physiological differences were more significant between H and CK groups, and more genes were found to be differentially expressed between these two groups, these DEGs were chosen for further functional analysis. To put these DEGs into different groups based on their potential functions, we mapped all these genes to the terms in the GO database. They fell into three functional groups, which are BP (biological process), CC (cellular components) and MF (molecular function) (Fig. 4). Each functional group contains a few sub-groups. Based on the number of DEGs present in each sub-group, these sub-groups were ranked. The top four are “metabolic process”, “catalytic activity”, “single organism process” and “single organism metabolic process”. It is also interesting to notice that all groups contain more up-regulated genes than down-regulated genes. This implies that plants adapt to drought stress mainly by up-regulating substantial drought-responsive genes, and the expression of these metabolic genes changed greatly to allow protective adaptations to occur in *O*.* taihangensis*.

### KEGG pathway analysis of the DEGs

**Fig. 5:**
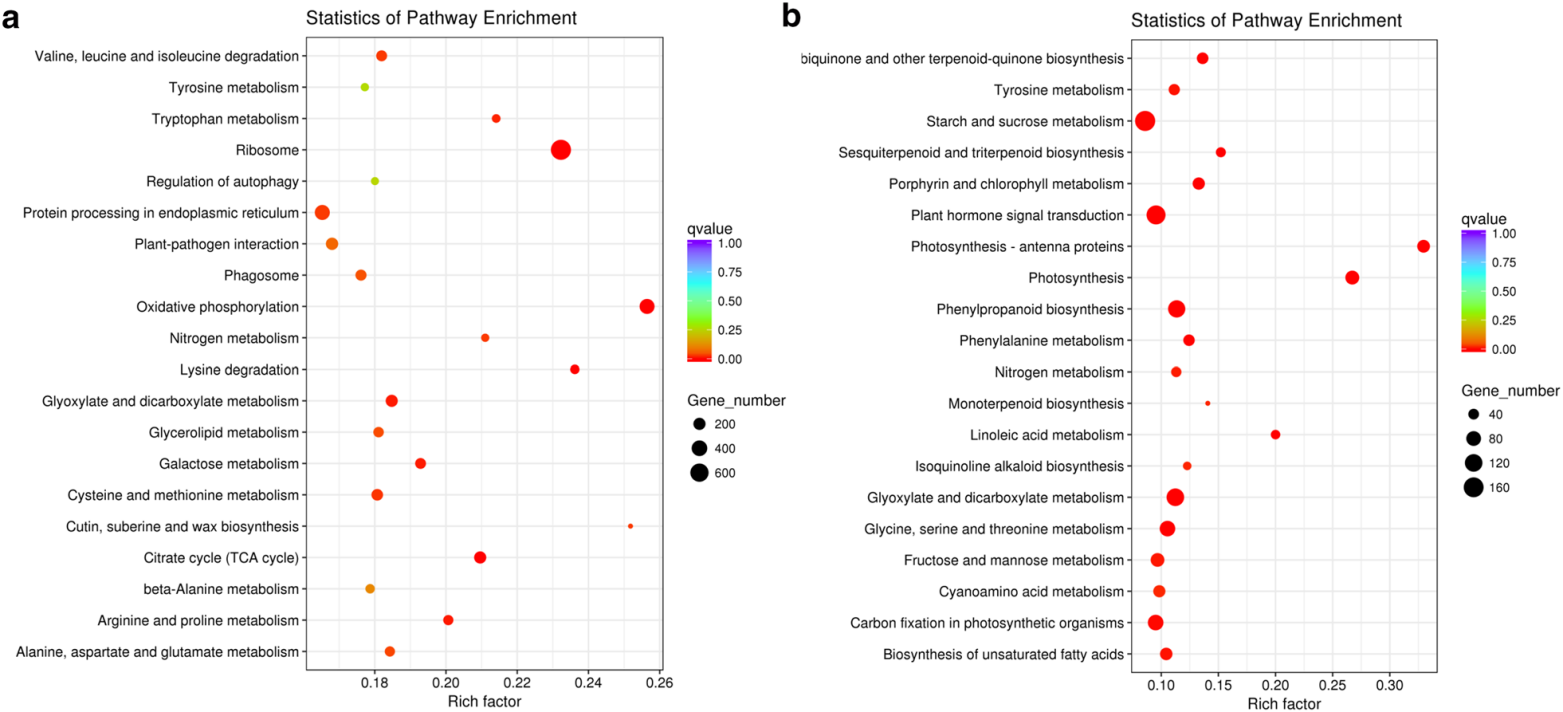
The KEGG analysis of the up-regulated (**a**) and down-regulated (**b**) DEGs identified by comparing H with CK. The pathways are listed along the y-axis. The X-axis indicates the rich factor. Red represents a high q value while blue represents a low q value

To further study the biological functions and interactions of genes, the DEGs were analyzed using the KEGG database. A total of 9573 up-regulated DEGs were annotated to 119 terms (Additional file 2: Table S2) and 4249 down-regulated DEGs were annotated to 118 terms (Additional file 3: Table S3). Among them, “ribosome” has the highest number of up-regulated DEGs, and most of the up-regulated DEGs were enriched primarily in the pathway of amino acid metabolism including cysteine and methionine metabolism, valine, leucine and isoleucine degradation, alanine, aspartate and glutamate metabolism, arginine and proline metabolism, beta-alanine metabolism, lysine degradation, tryptophan metabolism and tyrosine metabolism (Fig. 5a). Furthermore, we found that proline content increased dramatically (Fig. 1c), while proline biosynthetic genes involving in arginine and proline metabolism increased dramatically with the increase of drought stress, such as, Arg 1 (Arginase 1, Cluster-45536.56520), ODC1 (ornithine decarboxylase, Cluster-4897.2), OAT (ornithine-oxo-acid transaminase, Cluster-58171.0), PutA (proline utilization A, Cluster-45536.185548 and Cluster-45536.168614), proC (pyrroline-5-carboxylate reductase, Cluster-45536.70309) and P4HA (prolyl 4-hydroxylase, Cluster-13836.0 and Cluster-45536.160589). The specific information about these enzyme genes were shown in Additional file 4: Table S4. Besides, a large number of DEGs were enriched in the pathway involved in nitrogen metabolism. It is plausible that amino acids metabolism and nitrogen metabolism may have a prominent function in plant drought resistance.

In contrast, “starch and sucrose metabolism” has the highest number of down-regulated genes, which accounted for 12.04%. “Fructose and mannose metabolism”, “nitrogen metabolism” and amino acids metabolism is also significantly enriched in the group of high stress (Fig. 5b). This result indicates that plants may respond to the drought stress by synthesizing osmotic regulators, which are small neutral molecules stabilizing proteins and cell membranes to avoid cellular damage under stress. Moreover, some of the down-regulated genes are involved in other molecular functions, including “plant hormone signal transduction”, “carbon fixation in photosynthetic organisms” and “phenylpropanoid biosynthesis”.

### Analysis of drought-responsive transcription factors

**Fig. 6:**
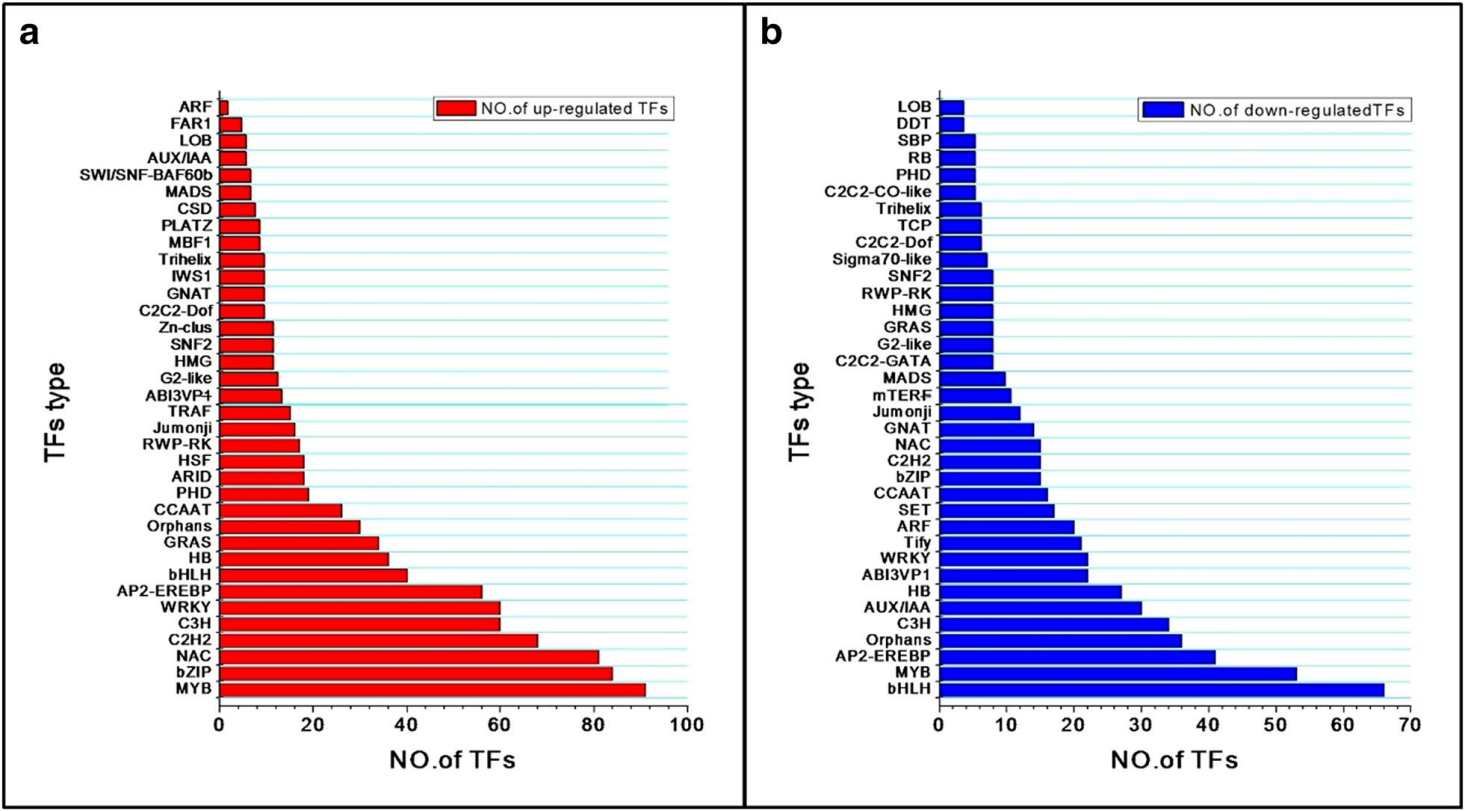
Classification of differentially expressed TFs (**a** the up-regulated TFs, **b** the down-regulated TFs) between H and CK. Abscissa indicates the number of genes assigned to a specific family; ordinate indicates the transcription factor types

Differentially expressed drought-stress responsive transcription factors (TFs) in *O. taihangensis* were identified by searching against the transcription factor database of *Arabidopsis* (https://plntfdb.bio.uni-potsdam.de). A total of 931 up-regulated TFs in H vs CK were identified and grouped into 36 families (Fig. 6a), including MYB (9.05%), bZIP (8.35%), NAC (8.052%), C2H2 (6.76%), WRKY (5.96%), and so on. In addition, 614 down-regulated TFs were categorized into 36 families, which include bHLH (10.02%), MYB (8.04%) and AP2-EREBP (6.22%). Moreover, a large number of TFs from the bZIP, C2H2, NAC, MADS families were significantly down-regulated in H group vs CK (Fig. 6b). These results indicate that TFs including MYB, bHLH, bZIP, NAC, WRKY, C2H2 and AP2-EREBP may contribute to plant drought tolerance. Interestingly, a total of 39 TFs were continuously up-regulated with the increase of drought stress, whereas 13 TFs were continuously down-regulated in H vs L and L vs CK, which may also be involved in plant resistance to drought stress. The specific information on the up-or down-regulated TFs were listed in Additional file 5: Table S5.

### qRT-PCR validation of the RNA-seq results

**Fig. 7:**
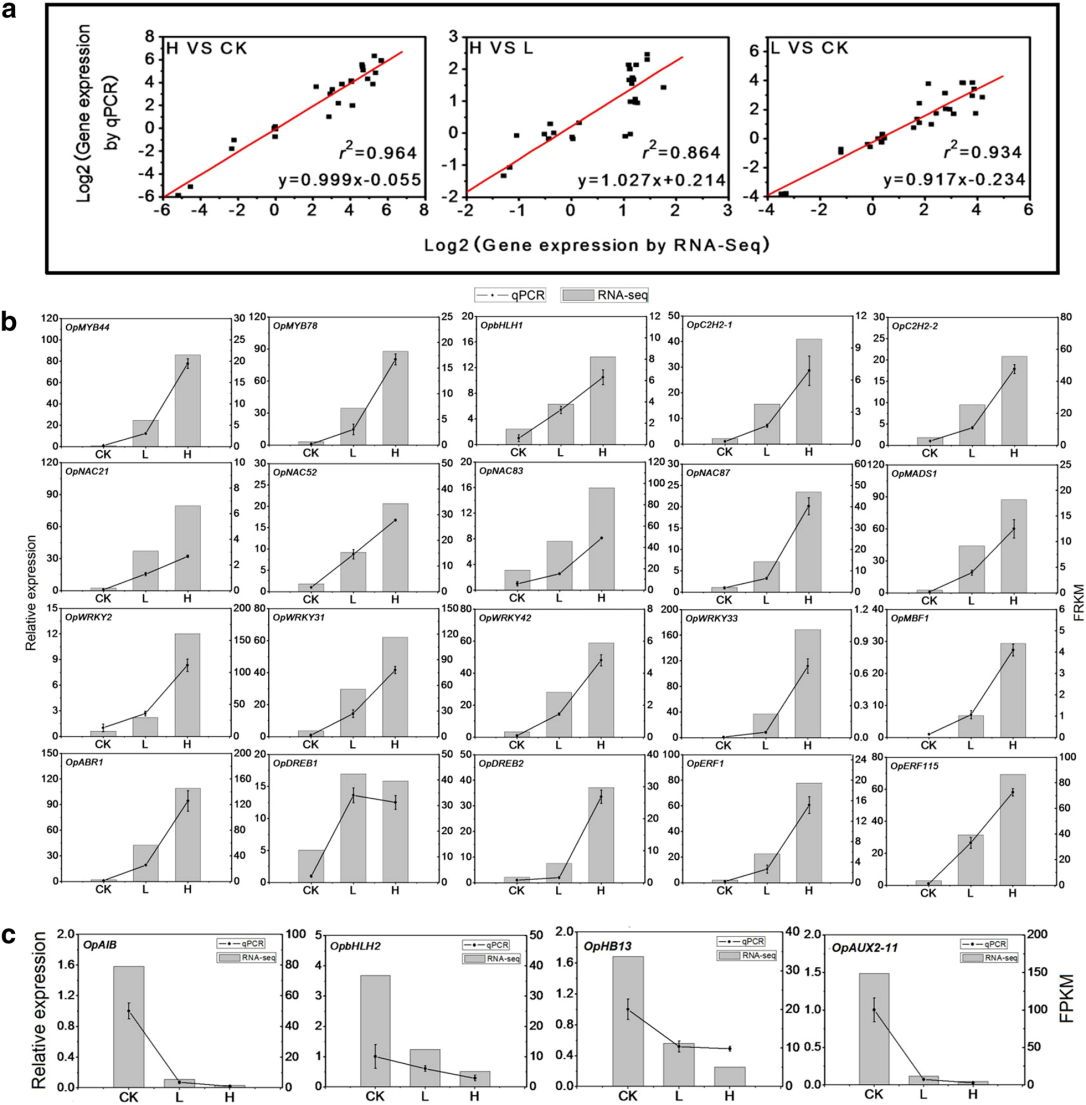
Quantitative RT-PCR analysis of the DEGs and TFs selected based on the RNA-Sequencing analysis results. **a** Linear correlation analyses were carried out using both the RNA-Seq data and the QRT-PCR data for the three comparison group (H vs CK, H vs L and L vs CK). The expression of both up-regulated (**b**) and down-regulated TFs (**c**) selected based on the RNA-Seq results in response to drought was analyzed using QRT-PCR. The left y-axis shows the relative gene expression levels analyzed by qPCR (black lines). The right y-axis indicates the corresponding expression data of RNA-seq (gray histogram). The x-axis represents the samples under different treatments. Bars represent SE (n = 3)

Furthermore, the expression of some drought regulatory genes, such as members from the MYB families, bHLH families, C2H2 families, NAC families, MADS-box families, WRKY families, AP2-EREBP families, AUX/IAA families and HB families, were examined using RT-qPCR. The results showed that most of the drought-responsive regulatory genes were up-regulated in plants under drought stress (Fig. 7b), except for OpDREB1 (Fig. 7c).

To verify the reliability of the RNA-Seq data, the expression pattern of 32 previously identified DEGs was further analyzed using qRT-PCR. Linear regression analyses were carried out for the three comparison group (H vs CK, H vs L and L vs CK) (Fig. 7a), and the overall correlation coefficients were R^2^ = 0.964, 0.864 and 0.934, respectively. High R^2^ values indicate a good correlation between the results of the qRT-PCR test and that of the RNA-seq differential expression analysis. This also suggests that the RNA-seq data is accurate enough to be used for subsequent analyses.

## Discussion

Plants respond differently to drought stress. They can cope adaptively via a series of physiological and metabolic responses including preventing, reducing or repairing damage to maintain a normal physiological state under water deficit [26]. For example, the completion of physiological and metabolic processes is achieved in plants under drought by osmotic regulation. Previous studies have shown that proline, an important osmotic regulator, plays an important role in plant resistance to drought [27]. The accumulation of proline is positively correlated with drought resistance of cultivars [28]. Highly drought-resistant cultivars can accumulate more proline, which can be used as a physiological index for identification of drought resistance [29]. In this study, the content of proline was increased gradually with the increase of PEG concentration (Fig. 1c), which indicates that *O. taihangensis* is able to decrease cellular osmotic potential, to maintain cell water content, and to improve plant drought resistance.

Plants are also able to tolerate drought stress by producing and accumulating high-affinity osmotic substances, such as betaine, proline, sucrose and fructose [30–32]. According to the KEGG analysis, most of the up-regulated DEGs were found to be involved in amino acid metabolic pathways and nitrogen metabolism, suggesting a critical role of amino acids and nitrogen-containing compounds to drought response in *O. taihangensis*.

Transcription factors are the main regulators of gene expression, and the activity of TFs often depends on developmental stages, exogenous stimuli, and/or the presence of co-regulatory proteins. Different TFs are employed for the expression regulation of different genes. Thus, TFs-based gene expression regulation allows plants to respond to the changes in their environment in a highly specific and flexible manner. In plants, different TF subfamilies or even members of the same subfamily might show different transcriptional regulation under various stress conditions [33]. A substantial amounts evidence suggests that members of the following TF subfamilies, such as AP2/EREBP, MYB, bHLH, WRKY, C2H2, MADS-box, NAC and AUX/IAA [34], are involved in plant drought-resistance response.

The MYB family is a large and functionally diverse TF family present in all eukaryotes. Members of this family can either act as positive or negative regulators in stress signal transduction in plants. Besides, MYB proteins are also promising targets for the generation of stress-tolerant crops. Previous studies have shown that the MYB TFs act through the ABA signaling cascade to control the opening of stomata and therefore water loss in rice and *Arabidopsis* [35, 36]. *AtMYB44* overexpression lines demonstrate increased salt tolerance by either activating the ROS scavenging system or inhibiting ROS formation. Besides, the transgenic plants were reported to be more tolerant under drought stress [37]. In this work, the expression of *OpMYB44*, a highly homologous gene of *AtMYB44 *(AT5G67300), was significantly induced in both L and H groups compared with CK. It makes perfect sense that *OpMYB44* shares a similar function with *ArabidopsisMYB44*, and that it may be critical to drought tolerance in *O. taihangensis*.

TFs of the bHLH family also play important roles in regulating responses to abiotic stress in plants. Recently, the expression of *AtAIB*, a gene of the bHLH family, was reported to be induced by drought stress, and the overexpression of this gene improved drought resistance in *A. thaliana* [38]. Similar results were obtained in *O. taihangensis*, *OpbHLH1 *was expressed to higher levels in both high- and low-stress groups in comparison to CK. In addition, the expression of *OpC2H2-1* and *OpC2H2-2 *was up-regulated in the L and H groups and was induced with the increase of drought-stress level. NAC TFs also play crucial roles in plant responses to abiotic stress and have been previously described to significantly improve drought tolerance in *Ammopiptanthus mongolicus* and *B. nivea* [39]. Four NAC genes (*OpNAC83, OpNAC21, OpNAC52, OpNAC83* and *OpNAC87*) in *O. taihangensis* were included in the analysis and found to be induced in plants under drought stress. WRKY proteins can regulate plant resistance by binding to the promoter regions of various stress-related genes. Members of the WRKY family were also found to be differentially expressed in *B. nivea* in drought treatment [40]. A similar pattern was observed in *O. taihangensis*, the expression of *OpWRKY2, OpWRKY31, OpWRKY33, OpWRKY42* were markedly induced by drought stress. Besides,the overexpression of *WRKY33* was sufficient to improve *Arabidopsis* NaCl tolerance [41].

According to recent genomic analyses, the AP2-domain protein family represents a large diverse family of plant-specific TFs, which includes the DREB subfamily (AP2 proteins regulating the expression of abiotic stress-responsive genes are referred to as DREBs), ERF subfamily (all the AP2-domain proteins, that are responsive to ethylene, were referred to as ERFs) and ABA repressor 1 (ABR1) [42, 43]. Interestingly, *RAP2.4B,* a homologous gene of the *OpDREB1*, was induced by cold, dehydration and osmotic stress. It plays an important role in the regulation of water homeostasis [44]. Overexpression lines of ERF1 (homologous gene of *OpERF1*) exhibit more tolerance to drought and salt stress, which suggests ERF1 contributes to plant tolerance to a variety of stresses including drought, salt, and heat stress [45]. ABR1 is strongly responsive to the ABA signal, and functions as a negative regulator of the ABA responses in *A. thaliana*. In our studies, the expression of *OpABR1* was induced by drought, and we found the mRNA level of *OpDREB1* was also induced by mild drought stress, but slightly decreased under severe drought stress. These results suggest that members of the AP2 protein family, OpABR1, OpDERB1 and OpERF1, have the function of regulating drought tolerance in *O. taihangensis*.

The expression of some genes, including *OpAIB* (ABA-inducible bHLH type transcription factor), *OpbHLH2, OpATHB13* and *OpAUX2-11 *(AUX/IAA transcription factor), were suppressed under drought stress (Fig. 7c). It is worth mentioning that the expression of *AtAIB*, a bHLH gene, was induced by ABA and PEG, suggesting that AtAIB functions as a transcription activator in the regulation of the ABA signal response in *Arabidopsis* [38]. This is contradictory to what we have found in *O. taihangensis*, where AIB was speculated to have an inhibitory role. This implies that bHLH proteins may behave in a species-specific manner. AtHB13, an HD-Zip protein, positively regulates cold stress by stabilizing cell membranes and inhibiting ice growth in *Arabidopsis* [46]. However, recent studies have shown that knockout mutants for *Athb13* displayed increased primary root length compared to wild-type seedlings, which suggests that this HD-Zip transcription factor is a negative regulator of early root growth [45]. In our studies, *OpHB13*, the highly homologous gene of *Arabidopsis HB1*, was strongly inhibited by drought stress. Therefore, we deduce that the down-regulated expression of *OpHB13* may be involved in drought resistance in *O. taihangensis*.

## Conclusions

It was demonstrated in this work that *O. taihangensis,* an endangered plant species in China, is able to adaptively cope with drought stress by changing its morphological and physiological traits. RNA-Seq differential gene expression analysis of *O. taihangensis* under drought allowed the identification of a large number of differentially expressed genes. These DEGs are able to either positively (up-regulated) or negatively (down-regulated) respond to drought. In addition, the up-regulated DEGs were mostly enriched in the pathway involved in amino acid metabolism, as well as TFs, which regulate the expression of drought-resistance genes to allow plants to avoid adverse effects caused by drought stress.

## Additional files

**Additional file 1: Table S1.** Primers used for RT-qPCR analysis of genes in* Opisthopappus taihangensis*.

**Additional file 2: Table S2.** The KEGG pathway analysis of up-regulated DEGs from HvsCK.

**Additional file 3: Table S3.** The KEGG pathway analysis of down-regulated DEGs from HvsCK.

**Additional file 4: Table S4.** The FPKM of proline biosynthetic genes in H group, L group and CK.

**Additional file 5: Table S5.** The analysis of all annotated transcription factors.

## Data Availability

All the data is contained in the manuscript.

## Abbreviations

PEG: polyethylene glycol treatments
RWC: relative water content maintenance
DEGs: differentially expressed genes
TFs: transcription factors
ABA: abscisic acid
JA: jasmonic acid
GA: gibberellic acid
ET: ethylene
BR: brassinosteroid
H group: high salinity
L group: low salinity
CK: control
FW: fresh weight
DW: dry weight
TW: turgid weight
FDR: false discovery rate
BP: biological process
CC: cellular components
MF: molecular function
