# Supplementary material for: Physiological and transcriptome analyses of Opisthopappus taihangensis in response to drought stress
Source: Cell Biosci. 2019 Jul 4;9:56. doi: 10.1186/s13578-019-0318-7 (PMC6611040; doi:10.1186/s13578-019-0318-7)
Supplement: Supplementary file 1 — Additional file 1: Table S1. Primers used for RT-qPCR analysis of genes in Opisthopappus taihangensis. [file 13578_2019_318_MOESM1_ESM.docx]

Table S1. Primers used for RT-qPCR analysis of genes in *Opisthopappus taihangensis*

| Gene name | Forward primer (5' to 3', top)/reverse primer  (5' to 3', bottom) | Gene ID |
| --- | --- | --- |
| *OpAIB* | GGCTTTCAAAATCAAGTAGG /GGTACCCAGGTGCCAAT | Cluster-45536.135662 |
| *OpbHLH2* | GGTGGTTGTACGACGGT /CCCCTTTTCTTTTCCCT | Cluster-45536.66365 |
| *OpAUX2-11* | GTGTCTCCGATGAAAACG /CACTTAAACATGCCTTGC | Cluster-45536.136041 |
| *OpHB13* | AACACCGCCCAACGA /CCAACAAACATGCTGCTA | Cluster-45536.125817 |
| *OpNAC52* | GAACCAGAGCCAGAAACG /CGCTGAGGTGGTAAGAGG | Cluster-45536.45819 |
| *OpNAC21* | TGCCTCCAGGTTTTCG /GGCTCGCACTTGTTTAGAT | Cluster-45536.29443 |
| *OpNAC83* | TGCTTCCATCATCCCTG /ACTTGACTTCCCTTGTGC | Cluster-45536.123050 |
| *OpNAC87* | GAATGCGGATGTTGGG /TTCGTTGTGGTTGTTGGT | Cluster-45536.31810 |
| *OpWRKY31* | CTCAACAGGTTTCGGTAGC /TGTCTTCACGACGGACAG | Cluster-45536.132680 |
| *OpWRKY33* | TCACATGATTCACAAGCTG /CGTGGTGGTGTAAGACGT | Cluster-7030.0 |
| *OpWRKY2* | TTCGGCATCGTTCGG /TCGGTCTCACCCTTCC | Cluster-45536.21213 |
| *OpWRKY42* | CGGCAGACGGTCACGAT /CCAAACCCGTATTTACATCA | Cluster-45536.131717 |
| *OpABR1* | CATTTGACACTGCGGAAGC /TGGGTGGTCGGATTTG | Cluster-45536.30387 |
| *OpERF1* | CAACCAAAATAACCACCTC /TCATGCAAAGACTCCCTC | Cluster-45536.209639 |
| *OpERF115* | TTTCTCCTCGGATTCACC /CGTGTTGGTTTTGTCGG | Cluster-45536.65308 |
| *OpMYB44* | CAAGAACCACTGGAACTCG /ATCCCATTACAGCTACTGACG | Cluster-45536.30687 |
| *OpMYB78* | TCTGACTTGACTGATTGCTAC /AACCGAGGGGCTGAT | Cluster-45536.229076 |
| *OpMADS1* | TAAGGTGGAACTGAAGCG /CCGTAAGCGAAACTGATAAT | Cluster-32697.0 |
| *OpbHLH1* | TGCTTGGCTGATGTCG /ATGGTGGTAATGTTGGTATG | Cluster-45536.204343 |
| *OpC2H2-1* | CCGAGCCGAGTCTTTG /GTCGCTCTATGTCTCCTCA | Cluster-60310.0 |
| *OpC2H2-2* | TAACCGTCAGTTTCCGTC /GGTCGCTCTATGCCTTCT | Cluster-45536.74985 |
| *OpDREB1* | CCCCAAAGCCCGTGAA /TGAGGCGAGCGTAGTCG | Cluster-45536.181237 |
| *OpDREB2* | GGCTGTTAAGGCGGCTAC /TCGGCAACTCAATAATCTCAC | Cluster-45536.10482 |
| *OpMBF1* | GGGGCTGAAGTGAGACA /CGGAACCGCTTTACCA | Cluster-45536.227784 |
| *Opactin* | TCCCACATTGTCCTCCT /TGTCAAAACAGCGTTCCT | Cluster-45536.132958 |
